# London Hospital: Surgical Cases under the Care of Mr. Maunder

**Published:** 1874-11-01

**Authors:** 


### LONDON HOSPITAL.—SURGICAL CASES.

### UNDER THE CARE OF MR. MAUNDER.

*From the London Lancet.*

1. *Compound Fracture of the Skull.*—Frederic B———, aged five, was admitted on June 17th, having fallen from a third-story window, a hight of about thirty feet.

On admission a horizontal laceration was found, about half an inch in length, above and to the inner side of the left frontal eminence, the wound extending down to the bone. At the bottom of the wound a large depressed fracture of the frontal bone could be felt, the depressed portion being considerably below its normal level and overlapped by the sound bone.

It was determined to raise the depressed and impacted bone. Hoffman's forceps were used to chip out a small crescent-shaped piece of the overhanging bone, so as to admit of the introduction of the elevator.

2. *Phosphorus Necrosis of the Lower Jaw.*—D. S———, aged thirty-three, had suffered eight months, during which time the whole of the body and large portions of the rami had become necrosed, and a thin shell of new bone was being modeled upon the original. With a raspatory introduced on all sides the dead bone was isolated from the living. The rami were divided with the saw and cutting forceps, and the whole mass removed in one piece through the mouth.

3. *Compound comminuted depressed Fracture of the Right Parietal Bone.*—P. M———, aged three, was injured by a fall. Hoffman's forceps were used to make room for the introduction of an elevator. The membranes and brain were found to be lacerated. The operation was performed on the 19th June, but on July 5th, almost complete hemiplegia having set in, Mr. Maunder evacuated an abscess in the right hemisphere. The full particulars of this case will be given when it is more complete.

4. *Strangulated Femoral Hernia.*—Margaret P———, aged sixty, was admitted June 23d, suffering from a strangulated femoral hernia on the right side. The protruded portion of bowel was about the size of a small walnut, and it had been strangulated twenty-four hours.

The patient having been anæsthetized, and taxis failing, an incision two inches in length was made at the inner side of the neck of the swelling. The constriction was discoved at the upper extremity of the crural canal. Some fibres of Gimbernat's ligament were divided, and the bowel returned without opening the sac. The wound was closed by three wire sutures.

5. *Multiple Cystic Tumor of the Ovary.*—Elizabeth M———, aged twenty-three, had observed an unnatural swelling of the abdomen about eight months ago. Has been tapped once, some months ago.

On June 24 ovariotomy was performed. There were extensive adhesions to the anterior abdominal wall, and also to the omentum, apparently radiating from the puncture. Bleeding vessels in the omentum were secured by fine catgut ligatures cut short. The pedicle, short and thin, was secured by a double whipcord ligature cut short, and the whole dropped back into the abdominal cavity, after the open ends of two large arteries on the surface of the pedicle had been seared by the actual cautery.

6. *Treatment of Exostosis by Subcutaneous Fracture.*—A girl about sixteen years old had a globular exostosis attached by a narrow stem to the lower part of the femur on the outer aspect, and near to the knee-joint. Mr. Maunder had frequently discussed the treatment of the case, and, among other things, suggested the feasibility of subcutaneous fracture and its possible consequences. But as the patient suffered from catarrh for some days, the operation was postponed until July 8th. Chloroform having been administered, the skin was first protected by a piece of chamois leather, and then the tumor, being seized with a pair of gas-fitter's pliers, was broken off with a jerk. Forty-eight hours afterwards some tenderness and swelling had resulted.

With the exception of Case 3, the patients are progressing most favorably. The only complaint of the ovarian case is that she is "tired of bed."

In suitable cases Mr. Maunder thinks that Hoffman's forceps should be used instead of the trephine, because sound bone is thus economized.

---

Dr. Q. C. Smith, (*Nashville Journal of Med. and Surg.*) considers the sub-nitrate of bismuth as a very efficient catarrh snuff. He says that when the disease only affects the nasal passages and frontal sinuses, it will often effect a cure if the disease is not of long standing. The mode of using is to take a pinch of the powder, and thrust it well up one nostril, closing the other one, and take several short whiffs, liberating a portion at each inspiration. Apply to each nostril in same way quite frequently.

---

Swallowing a Tool-Chest.—It is reported that in the different prisons of Paris there are five or six deaths every year from the effect of swallowing what is known as an "escape box." This remarkable box is made for the special accommodation of prisoners. It is of polished steel, about three inches long, and contains turn-screws, hammers, silk thread, and other implements necessary for escape. The box appears to be easily swallowed, but sometimes fails to reappear as intended, and the death of the victim is the result. But, when it does pass the bowels, the lucky prisoner is prepared to cut the thickest iron bars and set himself at liberty.

Typhoid Fever.—During the recent epidemic of typhoid fever at Lyons there occurred certain atmospheric changes of considerable collateral moment. The temperature rose suddenly, while the barometer experienced a heavy fall. Now, the falling of the barometer is always coincident with an increased discharge of the airs dissolved in water. This may be witnessed at such periods in the increased escape of marsh gas, and is exemplified in the operation of the common hubble-bubble pipe for smoking, so named. The ill-washed gutters of the streets and quays of Lyons emitted the most noisome exhalations (*des puantes emanations*). The quarter of the Bourse, the quays of the Rhone, both close to the public Lyceum, the Quai de Retz in especial, all abounding with dirt and stench, are successively implicated. Out of 900 boys at the Lyceum, 80 were laid up with typhoid fever; the institution, consequently, was closed, by the decision of the rector of the academy. This fever was characterized by evening axacerbations, and Dr. Bondet terms it, in certain cases, a regular abortive typhus. Without going into further details, it may be said that this epidemic of typhus, along with only too many of the same kind, points trumpet-tongued to the necessity of discontinuing the employment of sewers, which are no other than elongated cesspools, and the substitution of earth-closets along with the early removal of all animal and vegetable refuse, instead.—*La France Medicale.*

---

Ozone.—Dr. Lender ozonises chambers very successfully by means of a mixture of protoxide of maganese, or of the permanganate of potash and oxalic acid. Two spoonfuls of this powder, moistened with twice the amount of water, and a trifle more of water every two hours, emits ozone freely. Gold and silver, however, excepted, it oxidizes metals rapidly.—*Archivio di Medicina Chirurgia ed Igiene.*

